# Combined exposure to lifting and psychosocial strain at work and adverse pregnancy outcomes—A study in the Danish National Birth Cohort

**DOI:** 10.1371/journal.pone.0201842

**Published:** 2018-09-19

**Authors:** Camilla Sandal Sejbaek, Hans Bay, Ann Dyreborg Larsen, Petter Kristensen, Vivi Schlünssen, Anne-Marie Nybo Andersen, Jens Peter Bonde, Mette Juhl, Karin Sørig Hougaard

**Affiliations:** 1 National Research Centre for the Working Environment, Copenhagen, Denmark; 2 Department of Occupational Medicine and Epidemiology, National Institute of Occupational Health, Oslo, Norway; 3 Department of Public Health, Institute of Environment, Occupation and Health, Aarhus University, Aarhus, Denmark; 4 Department of Public Health, Section of Epidemiology, University of Copenhagen, Copenhagen, Denmark; 5 Department of Occupational and Environmental Medicine, Bispebjerg Hospital, Copenhagen, Denmark; 6 Faculty of Health and Technology, Department of Nutrition and Midwifery, Metropolitan University College, Copenhagen, Denmark; 7 Department of Public Health, Section of Environmental Health, University of Copenhagen, Copenhagen, Denmark; Centre Hospitalier Universitaire Vaudois, FRANCE

## Abstract

**Background:**

Previous studies have investigated physical and psychosocial job exposures separately in relation to foetal growth. We therefore investigated if occupational lifting and psychosocial job strain interact to affect foetal growth and gestational length. We hypothesised that heavy lifting and high job strain would increase the risk of impacted foetal growth (small or large for gestational age) and preterm birth.

**Methods:**

The cohort included 47,582 pregnancies from the Danish National Birth Cohort (1996–2002), where the woman was pregnant at 22 gestational weeks (GW), expected one child and worked ≥30 hours/week. Information on occupational lifting and psychosocial job strain was derived from an interview (16±3.0 GW). Data to calculate small and large for gestational age (SGA/LGA) and gestational length was retrieved from the Medical Birth Register. Interaction between lifting and job strain (Karasek’s model) was analysed by multinomial logistic regression.

**Results:**

Overall, the adjusted regression analysis showed statistically significant interaction between lifting and job strain for SGA and LGA. For each additional 250 kg lifted/day, high strain women (high Demand/low Control) had increased odds of giving birth to a LGA-child (OR = 1.15; 95% CI 1.06–1.26), whereas women in the active group (high Demand/high Control) had increased odds of giving birth to a SGA child (OR = 1.12; 95% CI 1.03–1.23). When women lifting ≤1000 kg/day were excluded in the sensitivity analyses the interaction between lifting and job strain became insignificant. No interaction of lifting and job strain was found for gestational length.

**Conclusions:**

The main findings may give some support to our hypothesis, as lifting in combination high with job strain increased the risk of giving birth to a LGA child. This finding was, however, not supported in the sensitivity analysis and no association of the interaction was found relative to gestational length.

## Background

In 2015, the employment rate was 72% for women in the reproductive age in Denmark (age 20–44 years) [1]. The impact of the working environment on pregnancy is therefore of major interest.

Psychosocial and physical (e.g. heavy lifting) strain are two common factors in the occupational setting. As individual factors, both have shown some association with adverse pregnancy outcomes. The few existing studies on psychosocial work exposures overall indicate a modest association between job strain and preterm birth and low birth weight although no firm conclusion could be reached [2]. However, general psychosocial stress has also been linked to abnormal glucose metabolism and increased blood glucose levels [3, 4], a predictor of children being born large for gestational age (LGA) to women without diagnosed diabetes [5]. A systematic review including papers from 1966 to 2010 does not indicate large effects of lifting at work on pregnancy [6]. Findings in later studies of high quality, however, indicate that there is a need for further study of this association [7–9].

Recently, high levels of physical activity at work were found to be associated with small for gestational age (SGA) and preterm birth, while no associations for adverse pregnancy outcomes were found for psychosocial stressors at work [10]. Exposures were assessed from a job exposure matrix. The interaction between the two exposures was not addressed, but the authors concluded that an understanding of such interaction and its relation to adverse birth outcomes is highly needed [10].

In Denmark, the official guideline for work during pregnancy recommends alleviation in case work tasks include heavy lifting or longer periods of walking and standing. Psychosocial strain should, however, mainly be considered when it co-occurs with physical strain [11], and co-occurrence of both exposures in the occupational setting will be common. To our knowledge, no studies have investigated if and how physical and psychosocial strain at work interact during pregnancy. There is, nonetheless, indications that combinations of exposures, such as poor mental working environment with maternal smoking or higher age, may enhance the risk of adverse pregnancy outcome [2].

### Aim

We aimed to investigate the effect of combined physical demands, operationalised by lifting, and psychosocial strain, operationalised by the job strain model, on foetal growth and gestational length.

We hypothesised that women in jobs with heavy lifting and high job strain would be at higher risk of impacted foetal growth (SGA or LGA) and gestational length compared to women in jobs with no lifting and low job strain. As a secondary hypothesis, we proposed that this would also be the case for women lifting combined with high psychosocial Demands.

## Method and design

### Study population

The study is based on the nation-wide Danish National Birth Cohort (DNBC) including 100,418 pregnancies between 1996 and 2002 [12]. The women were invited by their general practitioner at their first antenatal visit, if they intended to carry the pregnancy to term, lived in Denmark, and could complete a telephone interview in Danish. A 1^st^ trimester interview included a wide range of topics such as the women’s health, habits and medication, and physical and psychosocial working environment.

For the present study (S1 Fig) the women should have completed the first interview before GW 22 (average 17 GW ± 4 weeks) and be working (N = 62,852); be pregnant with their first singleton pregnancy in the DNBC (N = 57,558); have information on relevant exposures and outcomes (N = 54,977) and co-variables (N = 53,287); and work at least 30 hours per week. This resulted in a final study population of 47,582 pregnancies.

Permissions to use and store data were obtained from the DNBC and the Danish Data Protection Agency. Data was anonymised before they were accessed by Statistics Denmark. Our data were combined via the unique personal identification number given to all Danish citizens at birth. Subsequently, Statistics Denmark converted the personal identification number into an anonymised code, which allows the researcher to combine data, but not access to the unique personal identification number. The Danish legislation requires approval from the Ethical Committee only for use of human tissue; hence, no ethical approval was needed.

### Exposure

#### Lifting/physical working environment

In the first interview, the women were asked “*In your job*, *do you daily lift more than 20 kilos at a time*, *similar to a case of beer*?”. By an affirmative answer they were also asked: “*How many times a day do you do this*?”. Similar questions were asked for daily lifts of 11–20 kilos, which was compared to lifting “*less than a case of beer and more than a bucket of water*”. To construct a continuous exposure variable for daily lifting, heavy and medium lifts were assigned values of 22.5 and 15 kg/lift, respectively, and summarised for each woman in correspondence with previous studies from the DNBC [7, 8]. Women who did not report any daily lifting, heavy and medium lifts were categorised as lifting 0 kg per day (non-lifters).

#### Psychosocial working environment

**Fig 1:**
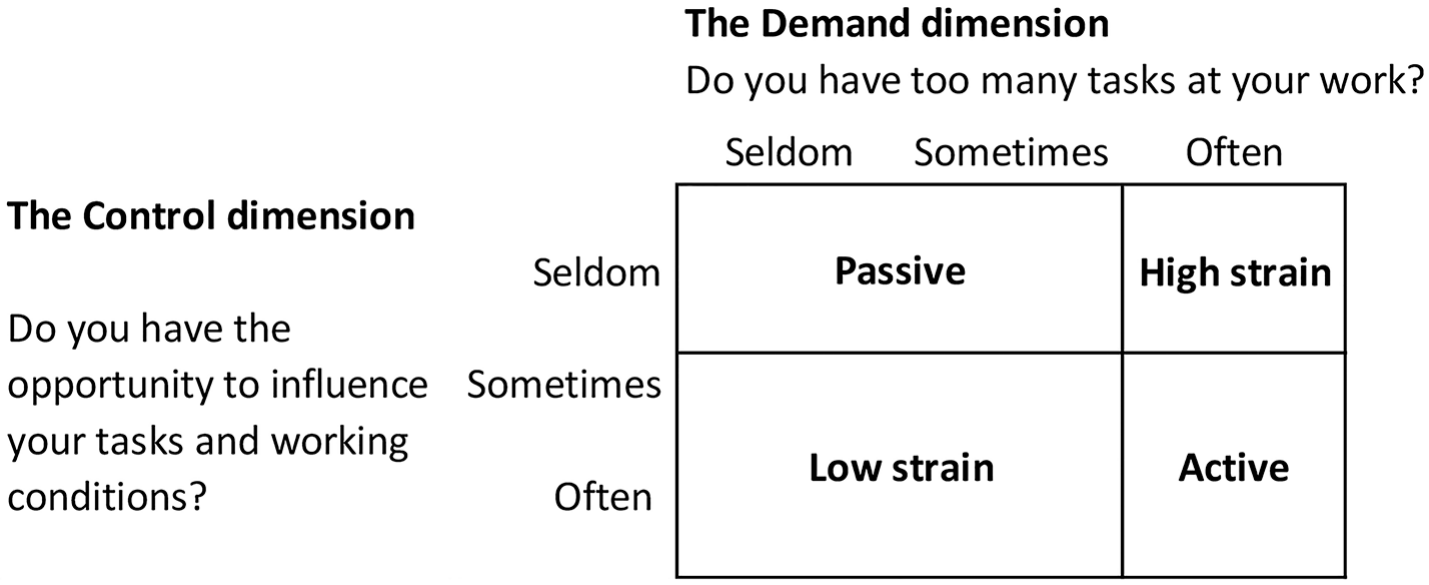
Job strain model. The combination of the Job Demand and Job Control dimensions as they are operationalised and applied in this study.

We used Karasek’s job strain model (Demand-Control Model) to assess the women’s psychosocial strain at work [13]. During the interview the women were asked these two questions “*Do you have too many tasks at your work*?” and *“Do you have the opportunity to influence your tasks and working conditions*?”, which were used to reflect Job Demand and Job Control, respectively. Each question had the response key of seldom, sometimes and often. The answers were combined in four categories in the job strain model (Fig 1), where their specific combination was used to maximise contrast in the exposure: 1. high job strain (high Demands, low Control), assumed to be the most stressful of the categories and associated with a higher risk of disease; 2. active (high Demands, high Control); 3. passive (low Demands, low Control); and 4. low strain (low Demands, high Control). To address our secondary hypothesis Job Demand and Job Control were also analysed separately.

### Outcome

Information on the first day of the woman’s last menstruation, the child’s date of birth and birth weight were retrieved from the Danish Medical Birth Register; linked via the mother’s personal identification number, which all Danish citizens receive at birth. Gestational age at birth was calculated as the number of days from the first day of the last menstrual period to the date of birth. Ultrasound examination for estimation of expected due date was not offered routinely to pregnant women during collection of DNBC baseline data.

To handle outliers and entry errors birth weights outside the interval of 125–6,000 g as well as birth weights outside the specified interval for each GW were excluded in accordance with previously established growth curves [14].

#### Gestational length

Deliveries after 44 completed GW were excluded from the study. A four-category outcome variable was generated: preterm (GW 22–36), early term (GW 37–38), term (GW 39–41, reference), and post term births (GW 42–44).

#### Foetal growth

Intrauterine growth was investigated as a three-category outcome variable (SGA, AGA [appropriate for gestational age] reference, and LGA [large for gestational age]). SGA was defined as the lowest 10^th^ percentile of children born within each gestational age for each sex [15, 16], while the 90^th^ percentile defined LGA children. The weight limits for SGA and LGA (separate for each sex) were based on Kiserud *et al*. [17] where data was retrieved from ultrasound measurements throughout low risk singleton pregnancies. This study did not report foetal weights estimates later than 40 completed GW [17]. Hence, information on SGA and LGA cut-offs as defined by Alexander et al. was applied for 41–44 GW (1996); i.e. the proportional weight increase/decrease for each week (41–44) relative to 40 GW was calcualted. The relevant weight increase/decrease was then added to the 40 GW weight reported by Kiserud *et al*. [17] for GW 41–44.

### Covariates

The potential confounders of the relationship between lifting, psychosocial working environment and gestational length or SGA and LGA were identified based on previous publications and depicted in directed acyclic graphs (DAGs) (S2 Fig).

Information on confounders was collected from the first interview early in pregnancy except for parity and maternal age at birth that were retrieved from the Danish Medical Birth Register. The list of confounders include: *Maternal age at birth* (< 25, 25–30, 31–35, >35 years old), *parity* (0, 1, ≥2), *maternal pre-pregnancy body mass index (BMI)* (15–18.4, 18.5–24.9, 25–29.9, 30–49.9 kg/m^2^), and during pregnancy *smoking* (no smoking, smoked not daily, daily smoking), *alcohol consumption* (unit alcohol per week: 0, <1, 1–2, and >2), *coffee intake* (yes/no), *physical exercise* (0, <3½ and ≥3½ hours/week), and *leisure time daily lifting* of more than 20 kg (yes /no). *Socio-economic position* (SEP) was constructed from self-reported job titles (higher education and/or work with management responsibilities, medium education, skilled work, unskilled work, and student [7].

### Statistical analysis

Three different approaches for the combination of lifting and psychosocial working conditions were conducted. Approach I: Lifting in combination with the four-category job strain model with low strain as reference. The multiplicative interaction term between lifting and the job strain model was included; approach II: Lifting in combination with Control with “often” as reference. The multiplicative interaction term between lifting and Control was included; and approach III: Lifting in combination with Demand with “seldom” as reference. The multiplicative interaction between lifting and Demand was included.

Multinomial logistic regression was conducted by the procedure Proc Logistic presenting odds ratios (OR) with 95% confidence intervals (CI). The likelihood ratio test was used to test the overall null-hypothesis, while the joint test was used to evaluate if the exposures were associated with the SGA and LGA compared to AGA.

In the multinomial logistic regression analyses, a three step procedure was applied. First, crude analyses were performed including only the two exposures and their interaction (Model 1); second, the analyses were adjusted for all the selected co-variables except SEP (due to a probable correlation between SEP and the exposures) (Model 2); and third, the analyses were adjusted for all co-variables (including SEP) (Model 3). The three step procedure was conducted for each of the approaches I-III.

To evaluate if the exposures were associated with preterm (GW 22–36), early term (GW37-38) and post term (GW 42–44) compared to term birth (GW 39–41) multinomial logistic regression was conducted with separate analyses for each of the three psychosocial approaches, as described above.

The results are presented within each stratum of the three approaches of psychosocial strain for the continuous exposure variable lifting as the risk of the outcome for each additional 250 kg lifted/day. The results are presented at the stratum of lifting at 250 kg for the different categories compared to the reference category for each of the three psychosocial approaches (job strain, Control and Demand, respectively) [18].

#### Sensitivity analysis

Lifting was included as a continuous variable in the main analyses; however, lifts might not exert similar influence all along the continuum. Therefore, we conducted sensitivity analyses by first, excluding women lifting more than 1000 kg/day; second, excluding women lifting more than 750 kg/day; and third, categorising the lifting variable into 6 categories (0–14 kg, 15–100, 101–200, 201–500 501–1000 and >1000 kg) in correspondence with previous DNBC studies [7, 8].

All analyses were conducted in SAS 9.4.

## Results

Table 1 presents maternal characteristics according to the 4 categories of the job strain model. Women in the passive group generally resembled those in the high strain group; while women in the active group tended to be more similar to women in the low strain group. The descriptive characteristics are, therefore, determined more by Job Control than Job Demand. Overall, women in the high strain group were younger, had children prior to enrolment in the DBNC, worked in unskilled jobs, and smoked during pregnancy. In addition, relatively more women in the high strain group did not drink alcohol and they had a higher BMI compared to women in the low strain group. Of the 47 582 eligible pregnancies, 3858 children were born SGA and 5913 children were born LGA; 2361 children were born preterm, 6586 early term, and 6237 post term.

**Table 1:** Characteristics of pregnant women working at least 30 hours/week according to the four categories of the job strain model. The Danish National Birth Cohort (N = 47 582).

Characteristics	Job strain model							
	High strain		Active		Passive		Low strain	
	N	%	N	%	N	%	N	%
	3913	8.2	11 244	23.6	4284	9.0	28 141	59.1
Maternal age at birth (years)								
<25	429	11.0	680	6.1	537	12.5	1864	6.6
25–29	1495	38.2	4090	36.4	1727	40.3	11 091	39.4
30–34	1461	37.3	4650	41.4	1473	34.4	10 990	39.1
> = 35	528	13.5	1824	16.2	547	12.8	4196	14.9
Gestational age at interview (weeks)								
<16	2000	51.1	5610	49.9	2271	53.0	14 437	51.3
16–21	1913	48.9	5634	50.1	2013	47.0	13 704	48.7
Parity								
0	1831	46.8	5623	50.0	2207	51.5	15 075	53.6
1	1428	36.5	4095	36.4	1514	35.3	9385	33.4
≥ 2	654	16.7	1526	13.6	563	13.1	3681	13.1
Socioeconomic position								
High education	256	6.5	1583	14.1	267	6.2	3283	11.7
Medium education	1075	27.5	3940	35.0	979	22.9	10 172	36.2
Skilled work	846	21.6	2493	22.2	1085	25.3	6353	22.6
Unskilled work	1521	38.9	2703	24.0	1661	38.8	6796	24.2
Student	215	5.5	525	4.7	292	6.8	1537	5.5
Smoking								
No	2628	67.2	8357	74.3	3008	70.2	21 920	77.9
Less than daily	517	13.2	1284	11.4	494	11.5	2905	10.3
Daily	768	19.6	1603	14.3	782	18.3	3316	11.8
Alcohol (unit/week)								
0	2345	60.0	6177	54.9	2429	56.7	14 982	53.2
<0	578	14.8	1703	15.2	695	16.2	4586	16.3
1–2	836	21.3	2842	25.3	1022	23.9	7350	26.1
>2	154	3.9	522	4.6	138	3.2	1223	4.4
Coffee								
0	2173	55.5	6106	54.3	2479	57.8	15 790	56.1
> 0	1740	44.5	5138	45.7	1805	42.2	12 351	43.9
Physical exercise (hours/week)								
None	2650	67.7	7038	62.6	2884	67.3	16 934	60.2
<3.5	997	25.5	3412	30.4	1145	26.7	9214	32.7
> = 3.5	266	6.8	794	7.1	255	6.0	1993	7.1
Leisure time lifting >20 kg								
Yes	290	7.4	789	7.0	260	6.1	1644	5.8
No	3623	92.6	10 455	93.0	4024	93.9	26 497	94.2
BMI (kg/m^2^)								
15–18.4	152	3.9	453	4.0	203	4.7	1120	4.0
18.5–24.9	2573	65.7	7844	69.8	2783	65.0	19 544	69.5
25–29.9	818	20.9	2095	18.6	900	21.0	5424	19.3
30–50	370	9.5	852	7.6	398	9.3	2053	7.3

BMI = Body Mass Index.

The average daily lifting burden was 186 kg for the 12 363 women in the group of lifters. Lifting was distributed differently across the job strain groups. In the high strain group, 42.6% of the women were daily lifters compared to only 22.1% in the low strain group. Women in the high strain group lifted on average 255 kg/day, while women in the low strain group on average lifted 100 kg less. The more Demand and the less Control the women experienced at work, the greater the proportion of daily lifters (Table 2). The results showed small differences between lifters and non-lifters within the four job strain groups in relation to both foetal growth and gestational length. Overall, the lifters seemed to experience more adverse outcomes compared to non-lifters, although the differences amounted to only 0.1–1.6 percentage points (Table 2).

**Table 2:** Combination of lifting and psychosocial strain groups and the prevalence of outcomes. The Danish National Birth Cohort (N = 47 582).

Psychosocial strain	N	Lifting			Foetal growth			Gestational length			
		Lifting status	% of each psychosocial strain group	Mean (kg)	SGA %	AGA %	LGA %	Preterm %	Early term %	Term %	Post term %
Job strain											
High	3913	Non-lifters^a^	57.4	-	8.5	79.8	11.8	4.8	14.4	68.0	12.7
		Lifters	42.6	255	9.1	77.9	13.0	5.6	16.4	66.1	11.8
Active	11 244	Non-lifters^a^	71.5	-	7.2	79.9	12.9	4.9	14.5	68.2	12.4
		Lifters	28.5	197	8.8	78.6	12.6	5.9	13.4	66.9	13.8
Passive	4284	Non-lifters^a^	70.1	-	8.6	79.8	11.6	4.8	14.6	67.2	13.4
		Lifters	29.9	223	9.8	78.0	12.2	5.1	14.4	66.0	14.5
Low	28 141	Non-lifters^a^	77.9	-	8.0	79.7	12.3	4.8	13.5	68.5	13.2
		Lifters	22.1	155	8.5	79.0	12.5	5.2	13.0	68.5	13.3
Demand											
Often	15 157	Non-lifters^a^	68.8	-	7.5	79.9	12.7	4.9	14.5	68.1	12.5
		Lifters	32.2	217	8.9	78.4	12.8	5.8	14.4	66.7	13.1
Sometimes	21 767	Non-lifters^a^	76.0	-	7.8	79.8	12.4	4.7	13.7	68.2	13.4
		Lifters	24.0	166	8.5	78.4	13.1	5.2	13.3	68.3	13.3
Seldom	10 685	Non-lifters^a^	78.5	-	8.5	79.6	12.0	4.9	13.5	68.7	12.9
		Lifters	21.3	168	9.1	79.9	11.0	5.1	13.3	67.6	14.0
Control											
Seldom	8197	Non-lifters^a^	64.0	-	8.6	79.8	11.7	4.8	14.5	67.6	13.1
		Lifters	36.0	241	9.4	77.9	12.7	5.4	15.5	66.1	13.0
Sometimes	14 772	Non-lifters^a^	73.6	-	7.7	80.4	11.9	4.8	14.0	68.1	13.0
		Lifters	26.4	178	8.5	79.5	12.0	5.4	13.7	68.1	12.8
Often	24 613	Non-lifters^a^	77.6	-	7.8	79.4	12.8	4.8	13.6	68.6	13.0
		Lifters	22.4	163	8.7	78.4	12.9	5.4	12.8	68.0	13.9

SGA = small for gestational age; AGA = appropriate for gestational age; LGA = large for gestational age.

^a^ Non-lifters: Included women lifting 0 kg/day and women who reported to lift heavy and medium lifts less than daily.

For foetal growth, the multiplicative interaction of lifting and psychosocial job strain was statistically significant in the crude analysis (approach I, Model 1: P = 0.04) and remained so after adjustment (Model 2 and 3: P = 0.003; Table 3). Women in the high strain group had an increased risk (Model 3, adjusted OR (aOR) = 1.15, 95% CI 1.06–1.26; Table 3) for having a LGA child for each additional 250 kg the women lifted/day. Women in the active group had an increased risk (Model 3, aOR = 1.12, 95% CI 1.03–1.23) for giving birth to a SGA child for each additional 250 kg lifted daily. On the other hand, no statistically significant increased odds were found for each of the psychosocial strain approaches within the stratum of lifting of 250 kg for foetal growth. Overall, differences in the odds for having a SGA or LGA child were statistically significant for lifting within the groups of psychosocial strain but not for psychosocial strain within the strata of lifting (250 kg).

**Table 3:** The multiplicative interaction between daily lifting and job strain model (approach I) on the odds ratio (OR) of giving birth to a child born small for gestational age (SGA) or large for gestational age (LGA) compared to appropriate for gestational age (AGA). The Danish National Birth Cohort (N = 47 582). The results are presented within the stratum of lifting at 250 kg and within the four strata of the job strain model. P-values for the multiplicative interaction between lifting and the job strain model in the three models: Model 1, P = 0.04; Model 2, P = 0.003; Model 3, P = 0.003.

	Lift within stratum of 250 kg and job strain = low (reference)						Within stratum of the job strain model							
							High strain		Active		Passive		Low strain	
	High strain		Active		Passive		Lift—risk at 250 kg increase		Lift—risk at 250 kg increase		Lift—risk at 250 kg increase		Lift—risk at 250 kg increase	
	OR	95% CI	OR	95% CI	OR	95% CI	OR	95% CI	OR	95% CI	OR	95% CI	OR	95% CI
Model 1														
SGA	1.07	0.92–1.24	1.00	0.89–1.04	1.15	0.99–1.34	1.05	0.94–1.17	**1.15**	**1.05–1.25**	1.12	1.00–1.25	1.06	0.97–1.15
LGA	1.08	0.95–1.22	1.03	0.92–1.15	0.93	0.80–1.08	**1.16**	**1.07–1.26**	0.99	0.91–1.08	0.97	0.85–1.10	1.01	0.93–1.08
Model 2^a^														
SGA	1.01	0.86–1.18	0.97	0.86–1.10	1.08	0.92–1.26	1.01	0.90–1.14	**1.13**	**1.04–1.24**	1.10	0.98–1.24	1.06	0.98–1.15
LGA	1.08	0.95–1.02	1.05	0.93–1.18	0.94	0.80–1.10	**1.15**	**1.06–1.26**	0.95	0.87–1.04	0.92	0.81–1.05	0.94	0.87–1.01
Model 3^b^														
SGA	0.99	0.85–1.16	0.98	0.86–1.11	1.06	0.90–1.24	1.01	0.90–1.13	**1.12**	**1.03–1.23**	1.09	0.97–1.23	1.05	0.96–1.14
LGA	1.08	0.95–1.23	1.05	0.93–1.18	0.94	0.80–1.10	**1.15**	**1.06–1.26**	0.95	0.87–1.04	0.93	0.81–1.06	0.94	0.87–1.02

Bold denotes significant OR: p < 0.05. 95% CI = 95% confidence interval.

^a^Adjusted for maternal age at birth, parity, alcohol, smoking, maternal body mass index, coffee, physical exercise, and leisure time daily lifting.

^b^Adjusted for maternal age at birth, parity, alcohol, smoking, maternal body mass index, coffee, physical exercise, leisure time daily lifting, and socioeconomic position.

For approach II, the interaction of lifting and Job Control was statistically insignificant in the crude analysis (Model 1: P = 0.14; Table 4) but significant in both of the adjusted models (Model 2 and 3: P = 0.01 and P = 0.02, respectively). For women responding to often have Control the adjusted odds of giving birth to a SGA child was 1.13 for each additional 250 kg lifted daily (Model 3, 95% CI 1.05–1.22, Table 4). Within the stratum of lifting 250 kg per day, women that sometimes experienced Control at work had a decreased odds of giving birth to a SGA child (Model 3, aOR = 0.86, 95% CI 0.76–0.98).

**Table 4:** The multiplicative interaction between daily lifting and Job Control (approach II) on the odds ratio (OR) of giving birth to a child born small for gestational age (SGA) or large for gestational age (LGA) compared to appropriate for gestational age (AGA). The Danish National Birth Cohort (N = 47 582). The results are presented within the stratum of lifting at 250 kg and within the three strata of Job Control. P-values for the multiplicative interaction between lifting and Control in the three models: Model 1, P = 0.14; Model 2, P = 0.01; Model 3, P = 0.02.

	Lift within stratum at 250 kg and Control = Often (reference)				Within stratum of Control					
					Seldom		Sometimes		Often	
	Seldom		Sometimes		Lift—risk at 250 kg increase		Lift—risk at 250 kg increase		Lift—risk at 250 kg increase	
	OR	95% CI	OR	95% CI	OR	95% CI	OR	95% CI	OR	95% CI
Model 1										
SGA	1.04	0.92–1.17	0.89	0.78–1.01	1.08	1.00–1.17	1.02	0.92–1.13	1.14	1.06–1.23
LGA	0.98	0.88–1.10	0.91	0.81–1.02	1.09	1.02–1.17	1.00	0.92–1.09	1.01	0.94–1.09
Model 2^a^										
SGA	0.98	0.87–1.10	**0.86**	**0.76–0.98**	1.05	0.97–1.14	1.00	0.90–1.11	**1.15**	**1.07–1.24**
LGA	1.00	0.89–1.23	0.94	0.83–1.05	1.07	0.99–1.14	0.95	0.87–1.04	0.94	0.87–1.02
Model 3^b^										
SGA	0.96	0.85–1.08	**0.86**	**0.76–0.98**	1.04	0.96–1.13	0.99	0.89–1.10	**1.13**	**1.05–1.22**
LGA	1.00	0.89–0.12	0.94	0.83–1.05	1.07	0.99–1.15	0.96	0.88–1.05	0.94	0.87–1.02

Bold denotes significant OR: p < 0.05. 95% CI = 95% confidence interval.

^a^ Adjusted for maternal age at birth, parity, alcohol, smoking, maternal body mass index, coffee, physical exercise, and leisure time daily lifting.

^b^ Adjusted for maternal age at birth, parity, alcohol, smoking, maternal body mass index, coffee, physical exercise, leisure time daily lifting, and socioeconomic position.

For approach III, the overall analyses of the interaction between lifting and Job Demand were insignificantly associated to SGA and LGA, irrespective of the co-variables included (0.24 ≤ P ≤ 0.54; Table 5).

**Table 5:** The multiplicative interaction between daily lifting and Job Demand (approach III) on odds ratio (OR) of giving birth to a child born small for gestational age (SGA) or large for gestational age (LGA) compared to appropriate for gestational age (AGA). The Danish National Birth Cohort (N = 47 582). The results are presented within the stratum of lifting at 250 kg and within the three strata of Job Demand. P-values for the multiplicative interaction between lifting and Control in the three models: Model 1, P = 0.54; Model 2, P = 0.24; Model 3, P = 0.25.

	Lift within stratum at 250 kg and Demand = Seldom (reference)				Within stratum of Demand					
					Often		Sometimes		Seldom	
	Often		Sometimes		Lift—risk at 250 kg increase		Lift—risk at 250 kg increase		Lift—risk at 250 kg increase	
	OR	95% CI	OR	95% CI	OR	95% CI	OR	95% CI	OR	95% CI
Model 1										
SGA	0.93	0.81–1.07	0.93	0.80–1.07	1.11	1.04–1.19	1.08	0.99–1.17	1.08	0.97–1.21
LGA	1.17	1.03–1.34	1.11	0.97–1.28	1.07	1.01–1.13	1.01	0.93–1.09	0.96	0.86–1.08
Model 2^a^										
SGA	0.92	0.80–1.06	0.95	0.82–1.09	1.09	1.02–1.17	1.07	0.98–1.17	1.08	0.97–1.21
LGA	1.19	1.03–1.36	1.11	0.96–1.28	1.03	0.97–1.10	0.94	0.87–1.02	0.91	0.80–1.03
Model 3^b^										
SGA	0.93	0.81–1.07	0.96	0.83–1.11	1.08	1.00–1.16	1.06	0.97–1.15	1.07	0.95–1.19
LGA	1.18	1.03–1.36	1.10	0.95–1.28	1.04	0.98–1.10	0.94	0.87–1.02	0.91	0.81–1.03

95% CI = 95% confidence interval

^a^ Adjusted for maternal age at birth, parity, alcohol, smoking, maternal body mass index, coffee, physical exercise, and leisure time daily lifting.

^b^ Adjusted for maternal age at birth, parity, alcohol, smoking, maternal body mass index, coffee, physical exercise, leisure time daily lifting, and socioeconomic position.

For gestational age, the analyses showed no statistical significance for the multiplicative interaction between lifting and any of the three approaches for the psychosocial working conditions (I-III), whether crude or adjusted (P > 0.18; data not shown).

In the sensitivity analyses, where lifting was categorised into 6 groups the interaction between lifting and the job strain model as well as between lifting and Control was no longer statistically significant, irrespective of adjustments (data not shown). In the stepwise removal of heavy lifters, including lifting ≤1000 kg/day or ≤750 kg/day, the interaction between lifting and the job strain model went from moderately statistically significant to non-significant (fully adjusted model ≤1000 kg lifted/day P for interaction = 0.23; and ≤750 lifted/days P for interaction = 0.84). A similar pattern was observed for the analyses of lifting and Control (fully adjusted model ≤1000 kg lifted/day P for interaction = 0.33; and ≤750 lifted/days P for interaction = 0.85).

## Discussion

This study showed lifting did interact with job strain in relation to delivering SGA or LGA children. These findings were, however, not supported in the sensitivity analysis. Lifting and job strain did not interact relative to gestational length.

We hypothesised that women in jobs with high physical demand (operationalised by lifting) and high psychosocial strain at work (operationalised by job strain) would be at risk for changes in foetal growth and gestational length compared to women with low physical and psychosocial job strain. This was partly supported by the analyses. Overall, the multiplicative interaction of lifting and job strain was statistically significantly associated with foetal growth, in the crude and adjusted analyses. We saw that within women in the high strain group lifting increased the risk for delivering a LGA child. As a secondary hypothesis, we proposed that the Demand dimension of the job strain model would drive this interaction. The lack of interaction between high Job Demands and lifting therefore went against our *a priori* secondary hypothesis.

Unexpectedly, within women in the active group, lifting was associated to the risk for delivering a SGA child. Furthermore, for women that often experienced Control at work, the odds of delivering a SGA child increased with the daily amount lifted. As women in the active group were defined as having Control sometimes or often at work, it could be speculated that being in Control drove this latter association. Overall, our findings suggest that Control may be the driving factor within the job strain model rather than Demand, at least when combined with lifting.

This was also reported in the meta-analysis by Madsen and colleagues; they showed in non-pregnancy studies that low Job Control was associated to clinical depression, while Job Demand was not [19]. This contrasts that for other non-pregnancy related outcomes (fatigue, musculoskeletal complaints and emotional well-being) the effects of high job strain seemed better explained by Job Demands (rather than Control) [20]. The latter study showed that Job Demand and Job Control were unevenly distributed among three groups of skill levels (combined by educational level and type of work, e.g., manual vs. non-manual), indicating that socioeconomic factors may be an underlying factor of the findings [20]. In our study, the differential effects of Control and Demand on foetal growth were not explained by SEP. The proportion of lifters was also similar within the Control and Demand categories with most lifters in the seldom category of Demand and the often category of Control.

Previous findings had indicated that the Control and the Demand dimensions may independently predict preterm delivery [21]. In addition to the quadrants of the job strain model [13] we therefore also analysed each of the dimensions separately. This also allowed us to investigate if the two simple questions for Demand and Control could provide a simple tool that in combination with the calculated burden of daily lifting would identify pregnant women at risk due to combination of exposures. Our results, however, did not show a straight forward association between the combination of lifting and psychosocial strain at work; hence, precluding their use as a simple tool to identify pregnant women at risk.

The sensitivity analyses did not confirm our main findings, i.e. the interactions vanished when women lifting heavy loads were excluded from the analyses. This indicates that the main findings could have occurred by chance. Alternatively, the association could have been driven by the women with heavy lifting burdens. This was, on the other hand, not supported by sensitivity analysis where the women were categorised into six groups according to the accumulated daily lifting burden. Women in the highest lifting category in combination with psychosocial strain at work did not present with a significant association to the adverse pregnancy outcomes. In their review, Palmer and colleagues [6] reported only small risks of adverse pregnancy outcomes due to lifting at work, but Palmer *et al*. [22] still recommended to make reasonable adjustments in late pregnancy due to potential problems for the pregnant women in coping with excessive physical work, such as heavy lifting. Future studies ought to specifically address interaction in pregnant women with high physical strain at work.

Runge *et al*. [9] found a dose-response relationship between a six-group lifting variable and preterm birth. We did not observe an association between the combined exposures and gestational length for any of the job strain groups, indicating while lifting in itself might be associated to preterm birth; while lifting in combination with job strain are not very likely to increase the risk for preterm birth further [9].

The strengths of our study include the large cohort where the interview was conducted early in pregnancy before the women knew the outcome of their pregnancy. The women worked in several different trades and represented all educational levels increasing the generalisability of the outcomes. Furthermore, women were only included if they worked at least 30 hours/week, as part time work probably increases recuperation from straining work factors. In Vrijkotte et al, high job strain and high physical workload were both statistically significantly associated with increased risk of giving birth to a SGA infant and of reduced birth weight, if the pregnant women worked more (but not less) than 32 hours/week [23].

Another strength is the use of the newly published foetal ultrasound growth charts by Kiserud *et al*. [17] to set the predefined weight limits for the 10^th^ and 90^th^ percentiles for SGA and LGA, respectively. Previous DNBC studies derived these cut points from birth weights of all children born into the DNBC [7, 24]. Preterm children are often smaller than the foetuses that continue in utero. The new cut points may therefore distinguish more correctly between SGA, AGA and LGA children.

In the adjustment for SEP we were aware of the risk of over-adjustment. SEP was constructed from self-reported job titles, which would to some degree reflect the women’s exposure to especially physical work factors, as jobs in the lower social classes (skilled and non-skilled occupations) are characterized by a higher degree of physical strain [25]. The DAG constructed while designing the study indicated that it was important to adjust for SEP. The analyses were therefore conducted in three steps, i.e. a crude followed by two adjusted analyses, without and with SEP, respectively. Inclusion of SEP did, however, not influence the associations. The interaction between lifting and psychosocial strain therefore seems to be quite robust in regards to SEP.

The operationalisation of the job strain model constitutes a weakness of the study. Only one question was used to reflect the Demand and the Control dimension, respectively. In our operationalisation of the available questions contrast was maximised. The available data did therefore not comprehend the full job strain model [26]. No studies have, to our knowledge, investigated the validity of the two questions used in our study. Other questions could have led to different grouping with more women in the high strain group, which would have increased the statistical power in the study [24]. Nonetheless, two other single-item measures for stress was previously compared to three more validated multi-item measures of perceived stress. The authors concluded the two single questions could be considered reliable to measure perceived stress and with a similar validity to the multi-item measures for stress [27].

We, furthermore, assumed that exposures remained constant throughout pregnancy, but most likely changes occurred, especially in highly exposed women [23, 28]. Danish guidelines for pregnant women relies on planning and adaptation of physical work tasks to allow for sufficient rest and variation as well as on technical aid [11]. Such action takes place only after disclosure of pregnancy. DNBC collected information on work exposures during the first or second trimester. Women interviewed early and before disclosing the pregnancy at the workplace could potentially report a higher exposure than women responding later in pregnancy, as the employer was informed and work tasks might have been adjusted—even if exposure in fact had been similar at the earlier time point. Furthermore, the earlier a woman disclosed her pregnancy the earlier work adjustments could have been implemented. It follows, that a later announcement of pregnancy would increase the risk of a higher cumulative exposure. Both cases could potentially have led to misclassification. Interestingly, Danish employers are not obligated to take specific actions related to psychosocial strain in pregnant women, unless the women are already exposed to physical strain [11].

Another potential source of misclassification is the reported number of daily lifts by the women or the weight assumed per lift by us (estimated 15 kg or 22.5 kg). The total daily loads ranged from 0 to 1,875 kg. Among Danish supermarket employees, supervisors have reported the average load lifted per workday to be 1,212 kg (SD 861 kg, range 0–6,030, in men and women combined) [29]. The maximum amount lifted in the present study might therefore appear high, taking into account that the women were pregnant. Information on lifting relied on self-report and may therefore be less reliable with regards to the number of kg lifted. However, women who reported to lift many heavy loads will probably lift relatively more than women who reported less or no lifting, the comparison may therefore still be valid.

The reported associations and interactions between physical and psychosocial work factors were far from simple, indicating that a prevention strategy is not straight forward. In future studies association between combined working factors and preterm birth and other negative pregnancy outcomes should be investigated further.

A cautious recommendation would be to limit the number of potentially adverse exposures during pregnancy. This recommendation supports the guidelines from the Danish Working Environment Authorities [11] who recommends that the working environment of the pregnant women are assessed as a whole when the women are exposed to high physical strain.

## Conclusion

In the present study, no association of the interaction between lifting and job strain at work was found relative to gestational length, but the interaction was associated to foetal growth in the main analysis. The finding that lifting in combination with high job strain increased the risk of giving birth to a LGA child may lend some support to our main hypothesis. The finding was, however, not supported in the sensitivity analyses. Our secondary hypothesis was not confirmed, as lifting combined with high Job Demands was not associated with pregnancy outcomes. Unexpectedly, within women in the active group, lifting was associated to the risk for delivering a SGA child. In future studies, it would therefore be interesting to investigate if the effect of lifting on foetal growth pattern is determined by the type of job strain during pregnancy. Furthermore, the present study included two, relatively simplistic, measures of a complex physical and psychosocial working environment, assessed at a single time point. To fully elucidate whether job exposures interact to increase the risk for adverse pregnancy outcomes, future studies are advised to apply a less reductionistic approach.

## Supporting information

S1 Fig Flowchart. The study population from the Danish National Birth Cohort, 1996–2002.
(TIF)

S2 Fig Directed acyclic graphs (DAG) of the relationship between the exposures and the outcomes. (TIF)
