# Supplementary figures and images for: Combined exposure to lifting and psychosocial strain at work and adverse pregnancy outcomes—A study in the Danish National Birth Cohort
Source: PLoS One. 2018 Sep 19;13(9):e0201842. doi: 10.1371/journal.pone.0201842 (PMC6145591; doi:10.1371/journal.pone.0201842)

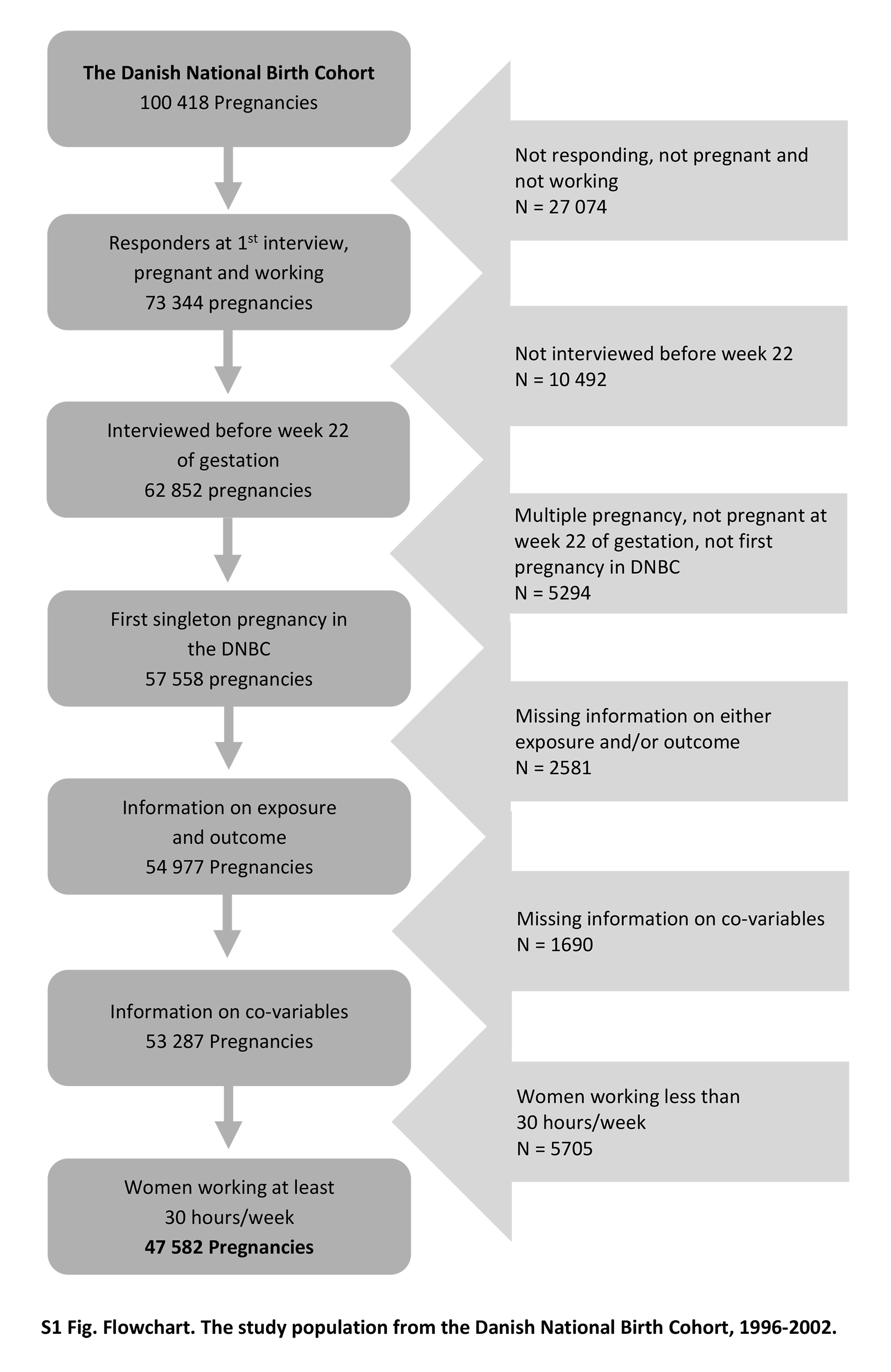

Supplement: S1 Fig — The study population from the Danish National Birth Cohort, 1996–2002. (TIF) [file pone.0201842.s001.tif]

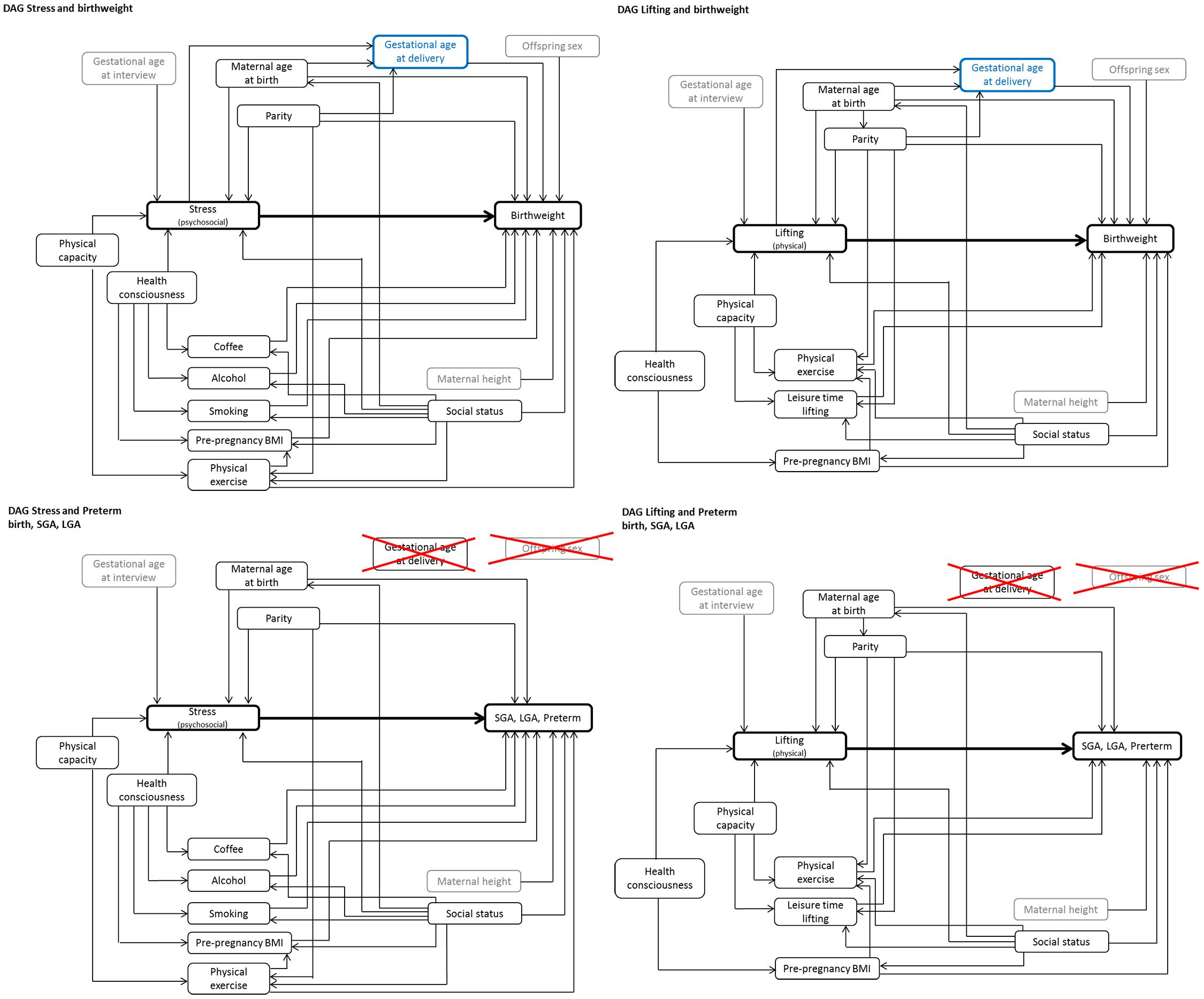

Supplement: S2 Fig — (TIF) [file pone.0201842.s002.tif]
